# Instant inactivation of aerosolized SARS-CoV-2 by dielectric filter discharge

**DOI:** 10.1371/journal.pone.0268049

**Published:** 2022-05-19

**Authors:** Ki Ho Baek, Donghwan Jang, Taeyoon Kim, Joo Young Park, Dojoon Kim, Sungweon Ryoo, Seunghun Lee

**Affiliations:** 1 Department of Nano-Bio Convergence, Korea Institute of Materials Science, Changwon, Republic of Korea; 2 Clinical Research Centre, Masan National Tuberculosis Hospital, Changwon, Republic of Korea; 3 Purunbit, Siheung-si, Gyeonggi-do, Republic of Korea; Waseda University: Waseda Daigaku, JAPAN

## Abstract

This study aimed to evaluate the instant inactivation effect of dielectric filter discharge (DFD) on severe acute respiratory syndrome coronavirus 2 (SARS-CoV-2) aerosols. The filter consisted of one layer of ZrO_2_ beads covered by aluminum mesh electrodes; this porous structure of DFD part generates filter-type surface discharge and reactive oxygen species. In a closed cylindrical chamber, DFD treated air flow containing SARS-CoV-2 aerosols, primarily composed of particle diameters of ≤ 1 μm. A polypropylene melt-blown filter collected the treated bioaerosols for inactivation analysis. Plaque and polymerase chain reaction assays showed that the aerosolized SARS-CoV-2 that passed through the filter were more than 99.84% inactivated with degradation of SARS-CoV-2 genes (*ORF1ab* and *E*). However, ozone exposure without DFD passage was not found to be effective for bioaerosol inactivation in plaque assay.

## Introduction

Severe acute respiratory syndrome coronavirus 2 (SARS-CoV-2) is a highly contagious virus that is responsible for the coronavirus disease 2019 (COVID-19) global pandemic [1]. Targeted vaccines and medicines are being developed worldwide, and practical strategies to prevent SARS-CoV-2 transmission and infection are still being implemented to alleviate the current public health situation. Some such strategies include wearing masks and “social distancing” to minimize SARS-CoV-2 spreading, as human-to-human transmission occurs through respiratory droplets [2–4]. Viruses are atomized by coughing or sneezing of an infected person, producing droplets (> 5 μm) and aerosols (< 5 μm) containing the virus [5]. Small aerosols can float in the air and carry viral contents up to several meters from their place of origin [3]; thus, some of these aerosols can eventually be inhaled directly by humans or can reach various surfaces. Moreover, SARS-CoV-2 can maintain its viability and infectivity for hours in aerosols and for days on various surfaces [6]. Therefore, the development of technologies that can directly inactivate SARS-CoV-2 aerosols, which are responsible for indoor transmission, is critical for minimizing viral transmission. Although several studies have been conducted to inactivate SARS-CoV-2 on surfaces [7, 8] and in solutions [9, 10], studies targeting SARS-CoV-2 aerosols remain scarce.

Establishing an appropriate ventilation system is an effective strategy for controlling bioaerosols. Although heating, ventilation, and air conditioning (HVAC) systems have been currently adopted to provide a healthy indoor environment [11], application of appropriate air purification technology is required to prevent ventilation system-related spreading of infectious aerosols [12]. It is more important to inactivate the virus to prevent secondary transmission in advance, rather than just promote physical filtration of bioaerosols using filters.

In this study, we developed a device for evaluating the inactivation effect of SARS-CoV-2 aerosols using atmospheric-pressure cold plasma as a model for the reaction portion of the ventilation system. In particular, an air-passable plasma filter that generates a dielectric filter discharge (DFD) at atmospheric pressure and room temperature was developed and applied as an ozone-based disinfection device. The generated ozone, including various reactive species, can naturally decompose in ambient air, representing minimal risk of secondary pollutant toxicity [13]. Importantly, previous studies have demonstrated the inactivating effect of plasma on SARS-CoV-2 and other viruses [7, 8, 14, 15], leading to viral RNA damage [16, 17]; thus, plasma may also hold virucidal potential against SARS-CoV-2 aerosols. Therefore, we aimed to evaluate the inactivation effect of DFD on aerosolized SARS-CoV-2 produced in extremely limited experimental environments.

## Materials and methods

### Dielectric filter discharge (DFD) generator

The configuration of the air-passable DFD generator is shown in S1 Fig. Zirconium oxide (ZrO_2_) beads (3 mm diameter), which form porous dielectric filter barriers, were covered on both sides with two punched aluminum electrodes (thickness: 0.5 mm, punching hole diameter: 2 mm, open area percentage: 58%). Sinusoidal voltage was applied to the electrodes at a maximum voltage of 2.5 kV and a frequency of 34 kHz. The continuous high voltage oscillation between the two aluminum electrodes can generate reactive oxygen species, such as ozone, through a localized electric field at the contact points of the beads and aluminum electrodes. In the 3 mm gap between the aluminum electrodes, aerosols can react with the reactive oxygen species, electrons, and electric fields. Unlike conventional ozone disinfection methods, DFD can apply conditions under which air and bioaerosols pass through the plasma zone.

### Plasma reaction chamber

**Fig 1:**
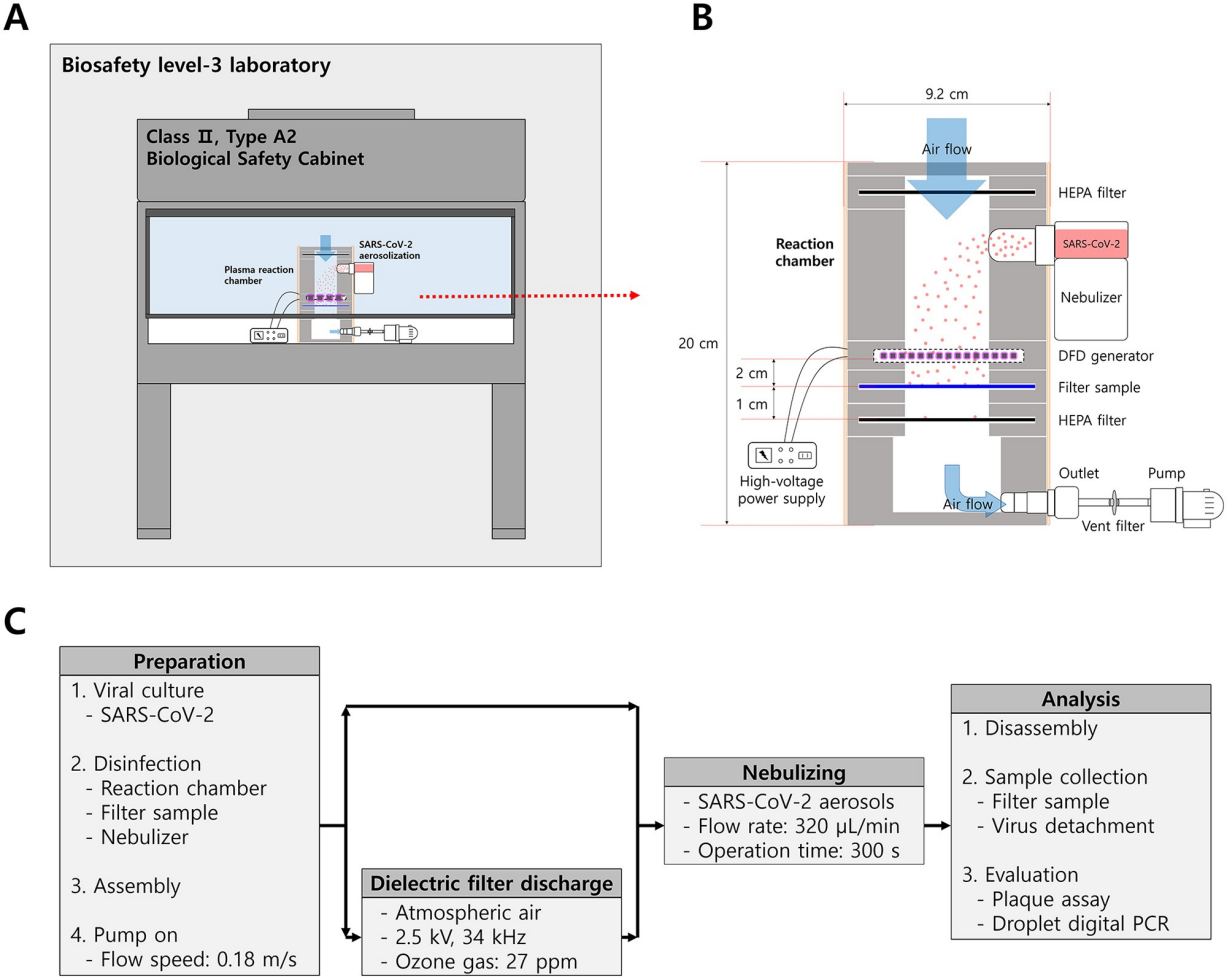
Experimental setup. Schematic diagram (not correctly scaled) of the (A) experimental environment and (B) plasma reaction chamber. (C) Flowchart of the experimental process.

Considering that the experiments using SARS-CoV-2 aerosols are harmful and limited, all experiments were conducted in a Class II Type A2 biological safety cabinet (BSC) located in a biosafety level 3 laboratory (Fig 1A). A closed-type cylindrical plasma reaction chamber was built on a portable scale, and all of the equipment used in the experiment was installed in the BSC. The main components of the reaction chamber included the DFD generator, high-voltage power supply, vibrating nebulizer (HL100A; Health & Life Co., New Taipei City, Taiwan), filter sample (7 cm diameter; CNTUS-SUNGJIN Co., Busan, Korea), high-efficiency particulate air (HEPA) filter, vent filter (polytetrafluoroethylene 0.22 μm membrane; GVS Life Sciences, Gyeonggi-do, Korea), and a pump (Fig 1B). HEPA filters were installed at the air inlet and outlet of the chamber to prevent the outflow of bioaerosols. The filter sample used to collect bioaerosols was a polypropylene melt-blown filter with a filtration efficiency of approximately 99.95% for 0.3 μm particles, which was disinfected using ultraviolet light for 30 min on both sides before the experiment. The chamber parts were assembled in the order of HEPA filter—nebulizer—DFD generator—filter sample—HEPA filter—pumping part. Each component was sealed by screw fastening type. The chamber stood vertically and the pumping part was placed at the bottom. The flow speed in the reaction chamber was 0.18 m/s, which was measured using a fan flow meter (AR856, Intell Instruments^™^ Plus, China). The flow speed at the space between the beads was 1–2 m/s, which was calculated using simulation software (GeoDict 2022, Math2Market GmbH, Kaiserslautern, Germany). The estimated residence time of aerosols in the plasma zone was 1.5–3 ms. The pressure loss at the flow speed of 0.18 m/s was 4.8 Pa, which was measured using a 100 × 100 mm size DFD generator that had the same internal structure as the DFD reactor. The simulation showed a pressure loss of 5.3 Pa at 0.18 m/s. The porosity at the maximum packing density was 42% when the volume of aluminum mesh was included.

### SARS-CoV-2 preparation

Vero 76 cells (CRL-1587; American Type Culture Collection, Manassas, VA, USA) were used for the propagation and viral infectivity assays for SARS-CoV-2 (NCCP 43326; National Culture Collection for Pathogens, Cheongju, Korea). Cells were incubated at 37°C with 5% CO_2_ in complete Dulbecco’s Modified Eagle Medium (DMEM; Gibco, Waltham, MA, USA) supplemented with 10% fetal bovine serum (FBS, Gibco) and 1% penicillin-streptomycin (Gibco). SARS-CoV-2 in vial stock was added to 80–90% confluency Vero 76 cells (75T flask) and adsorbed for 1 h at 37°C in a 5% CO_2_ incubator. Following adsorption, DMEM containing 2% FBS was added to the cells and the flasks were incubated for 72 h at 37°C in a 5% CO_2_ incubator until cytopathic effects were observed (S2 Fig). Virus-containing media were collected after centrifugation at 2,000 × *g* for 20 min (Allegra X-15R; Beckman Coulter, Fullerton, CA, USA), and the virus titer was approximately 2.87 × 10^6^ plaque-forming units (PFU)/mL.

### SARS-CoV-2 inactivation testing procedure

Fig 1C shows the experimental process of the control and treatment groups. The disinfected filter sample was assembled in a frame, and an air pump was operated to form a constant flow speed (0.18 m/s) in the reaction chamber. DFD was performed and stabilized according to the aforementioned conditions. SARS-CoV-2 aerosols were sprayed using the nebulizer at a flow rate of 320 μL/min for 300 s. A diffusion dryer that reduces the moisture of aerosols was not used because of the difficulties in operation and maintenance of the dryer components containing high-risk viruses. The aerosols passed through the plasma generation zone and were collected in a filter sample. The distance between DFD and the filter sample was 2 cm. The residence time of aerosols in the DFD zone was 16 ms, which was calculated by dividing the punched electrode gap by the flow speed. The same conditions without DFD were used for the control group. In addition, in order to evaluate the virucidal effect of surface ozone treatment under the condition that the aerosol does not pass through DFD zone, the SARS-CoV-2 aerosol was first sprayed for 300 s and then the DFD was operated for 300 s. Immediately after treatment, the filter was transferred to a 50 mL tube containing 10 mL of DMEM containing 2% FBS, and shaken for 2 min using vortex mixer to separate SARS-CoV-2. The particle size of the aerosols sprayed into the chamber was analyzed using a scanning mobility particle sizer. Considering the risk of the experiment, only the culture medium (DMEM containing 2% FBS) that did not contain the SARS-CoV-2 was used for particle size assessment.

### SARS-CoV-2 plaque assay

Plaque assay, which is the gold standard for the direct quantification of infectious viruses [18], was performed for SARS-CoV-2 as previously described [19]. Vero 76 cells were seeded in 6-well culture plates 24 h before infection in the presence of 10-fold serial dilutions of viral supernatants for 1 h. The infected cells were washed three times with phosphate buffered saline and cultured in 1% low-melting point agarose and 2% FBS-containing cell culture media in a 5% CO2 incubator at 37°C for 72 h. After aspirating the solid overlay from each well, the cells were fixed with 4% paraformaldehyde for 1 h at room temperature and stained with 0.5% crystal violet solution for 15 min (S2 Fig). The number of plaques were observed using a white-light transilluminator and expressed as log PFU/cm^2^.

### Viral RNA extraction and detection of SARS-CoV-2

After nebulization, viral RNA from the virus-collecting media was extracted using a QIAamp Viral RNA Mini Kit (Qiagen, Hilden, Germany). cDNA templates were synthesized using the iScript cDNA Synthesis Kit (Bio-Rad, Hercules, CA, USA) and amplified using the probe droplet digital PCR (ddPCR)-based method. To couple the two probes into a single ddPCR reaction, we designed primers and probes targeting two different genes of the SARS-CoV-2 genome using Primer3Plus [20] and the complete genome sequence (NCBI Reference Sequence: NC_045512.2). The sequences targeted for *ORF1ab* and *E* detection are shown in Table 1. Each probe was labelled at the 5′ end using fluorescein (FAM) dye.

**Table 1:** Sequences of the droplet digital PCR primers and probes used for detection of SARS-CoV-2 genes.

**Oligonucleotides**	**Sequence**
*ORF1ab* forward	5’-CTGAGCATAGTCTTGCCGAA-3’
*ORF1ab* reverse	5’-TCGGAACCTTCTCCAACAAC-3’
*ORF1ab* probe^a^	5’-[FAM]CCTATTGGGTTCCACGTGCTAGCGC-3’
*E* forward	5’-TTCGTTTCGGAAGAGACAGG-3’
*E* reverse	5’-AGACCAGAAGATCAGGAACTC-3’
*E* probe^a^	5’-[FAM]CTTCGATTGTGTGCGTACTGCTGC-3’

^a^Fluorescein (FAM)-tagged probes were used.

For ddPCR, a QX200 ddPCR System (Bio-Rad) was used according to the manufacturer’s instructions. The reaction was performed at a final volume of 20 μL, containing 10 μL of 2 × ddPCR Supermix for probes (no dUTP) (Bio-Rad), SARS-CoV-2 *ORF1ab* and *E* primers/probes with a final concentration of 900/250 nM, respectively, 3 μL of sample cDNA, and H_2_O to reach the final volume. The reaction mixture was placed in the sample well of a DG8 cartridge (Bio-Rad). A volume of 70 μL of Droplet Generation Oil for Probes (Bio-Rad) was loaded into the oil well and droplets were formed in the droplet generator (Bio-Rad). After processing, the droplets were transferred to a 96-well PCR plate (Bio-Rad). PCR amplification was performed on a C1000 Touch Thermal Cycler (Bio-Rad) with the following thermal profile: 95°C for 10 min, 40 cycles of 94°C for 30 s and 64°C for 1 min (ramp 2°C/s), on cycle at 98°C for 10 min, and ending at 4°C. After amplification, the plate was loaded onto an automatic droplet reader (Bio-Rad). QuantaSoft software was used to count the PCR-positive and -negative droplets to provide absolute quantification of the target DNA. Quantification of each target was expressed as copies number/μL of reaction.

### Statistical analysis

All experiments were performed in triplicate. Statistical analysis was performed using SAS software (version 9.4; SAS Institute Inc., Cary, NC, USA). Student’s *t*-test was used to assess differences between the groups, with *p* < 0.05 being considered significant.

## Results and discussion

### Physicochemical properties of aerosol and DFD

**Fig 2:**
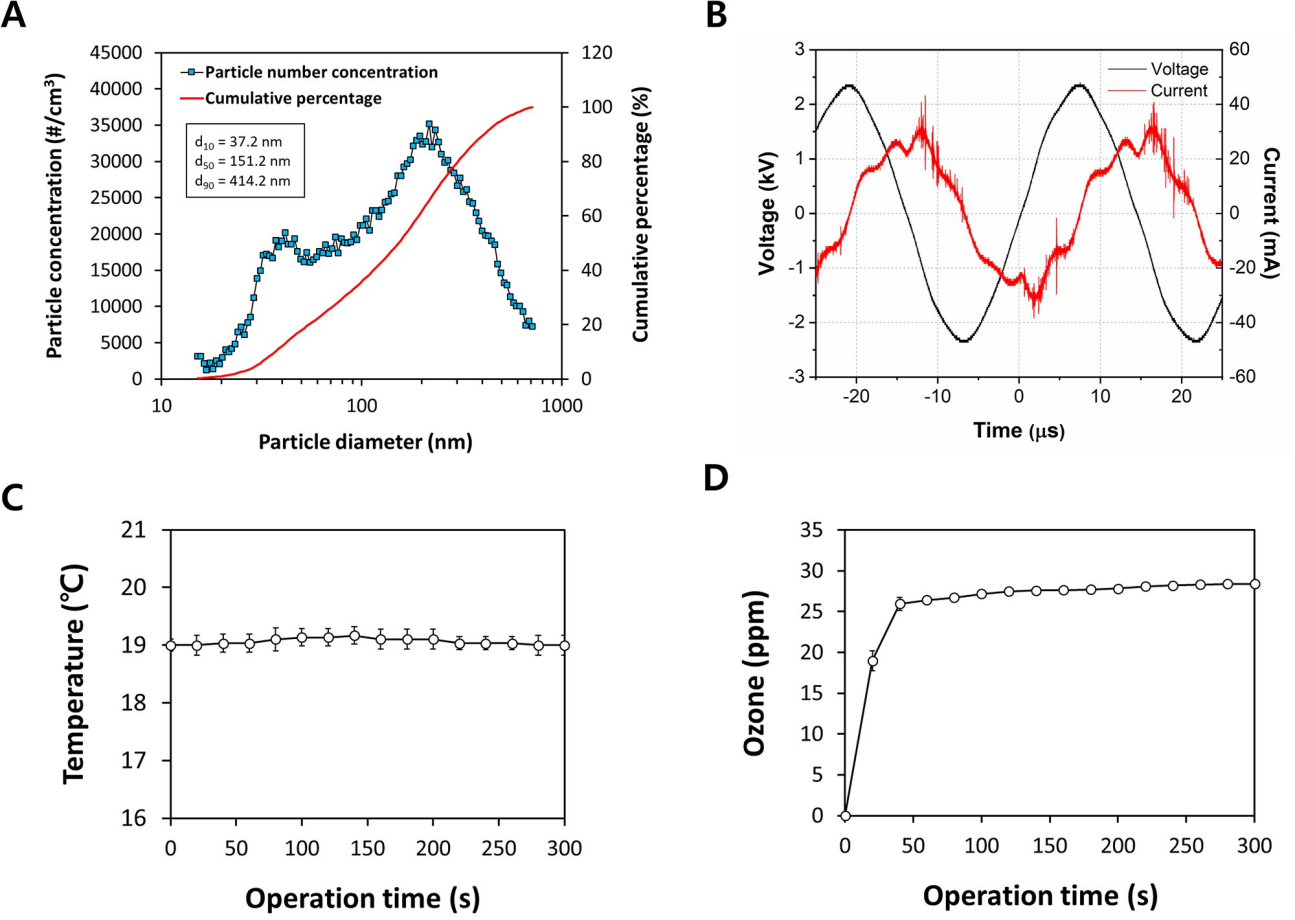
Physicochemical properties of the aerosol particles and dielectric filter discharge (DFD). (A) Size distribution of aerosol particles during nebulization. (B) Voltage and current waveforms, (C) temperature, and (D) ozone generation during DFD in a plasma reaction chamber. Considering the risk of the experiment, only culture medium without SARS-CoV-2 was used for the particle and system assessment.

Fig 2A shows the particle size distribution of aerosols sprayed into the chamber, of which the ratio of 15.1–414.2 nm particles was approximately 90%. These fine particles (≤ 1.0 μm) can evaporate within a few milliseconds, becoming virus-containing “droplet nuclei” and spreading over a wide area of air [2]. The air-passable DFD generator showed electrical characteristics (Fig 2B), which can be observed in the general dielectric barrier discharge [21–23]. The discharge remained stable even when the media aerosols passed through the discharge area. In addition, the temperature in the reaction chamber remained constant at 19.1 ± 0.06°C (*n* = 3) when sprayed for 300 s with DFD plasma (Fig 2C). The ozone concentration in the reaction chamber, influenced by DFD and airflow, increased to 27.0 ± 2.3 ppm (*n* = 3) within 20 s and was maintained (Fig 2D). In a similar system, ozone can be decomposed according to the discharge time when the gas temperature increases [24]. In this study, there was no increase in temperature due to discharge, and the ozone concentration remained constant. Overall, it was verified that non-thermal plasma can be uniformly produced and applied when the aerosols pass through the DFD generator.

### Inactivation of SARS-CoV-2 aerosols by DFD

**Fig 3:**
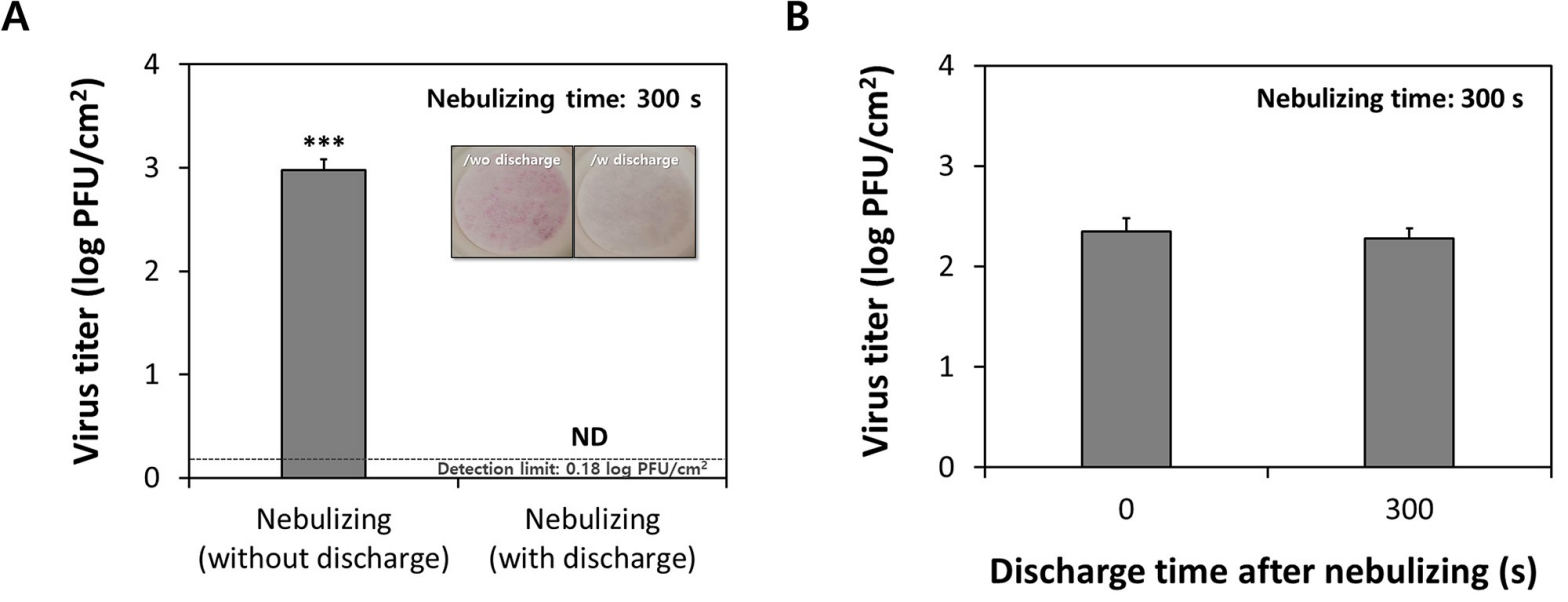
Inactivation of SARS-CoV-2 by DFD plasma. Infection of untreated and plasma-treated SARS-CoV-2 on Vero 76 cells. (A) SARS-CoV-2 aerosols were passed through the plasma generation area and were sprayed on the entire surface of the filter sample for 300 s. Inset: Visual aspects of filter samples according to plasma discharge during SARS-CoV-2 aerosol injection. (B) Plasma was applied to the filter-attached SARS-CoV-2 for 300 s after the virus solution was completely sprayed. Error bars indicate standard deviation (*n* = 3). ND: not detected (detection limit: 0.18 log PFU/cm^2^ filter). ***p < 0.001 by Student’s *t*-test.

The DFD plasma was found to induce inactivation of SARS-CoV-2 aerosols by more than 99.84% compared with the control group (Fig 3A), reaching an undetectable level (detection limit: 0.18 log PFU/cm^2^ filter) (*p* < 0.001). However, when DFD was performed for 300 s after the SARS-CoV-2 aerosol was completely sprayed, there was no significant inactivation effect on the virus attached to the filter sample (Fig 3B). This study excluded the analysis and interpretation of aerosol flow in terms of hydrodynamics, but we confirmed that bioaerosols passing through the DFD area could have a significant impact on viral inactivation. Ozone is a powerful oxidant that induces oxidative stress in living organisms, with verified antibacterial [25], antiviral [26], and antifungal [27] effects. Recently, ozone was shown to have an inactivation effect on human coronavirus (HCoV-229E), as a surrogate for SARS-CoV-2, on the surface of masks [8]. Chen *et al*. [7] also demonstrated that Ar-fed cold atmospheric plasma treatment is effective in deactivating SARS-related coronavirus 2 (isolate: USA-WA1/2020) on plastic, metal, cardboard, and leather surfaces, and thus has the potential to prevent virus transmission and infection over a wide range of materials. Herein, we evaluated the inactivation effect of DFD by generating SARS-CoV-2 aerosols that may occur in real environments and verified that DFD is effective for SARS-CoV-2 inactivation at an ozone concentration of approximately 27 ppm (Figs 2D and 3A). This is a relatively low ozone concentration compared to that of a previous study [28], which indicated that discharge conditions with approximately 870 ± 40 ppm of ozone eliminated the cytopathic effect of aerosolized SARS-CoV-2. Therefore, ozone may have acted as a secondary reagent in inactivating the bioaerosols in our study. SARS-CoV-2 aerosols can react with various reactive species as they passed through the plasma area in real-time (Fig 1B). In addition to the direct reaction of ozone to organisms, there is a possibility that various short-lived radicals can contribute to the inactivation of the virus contained in aerosols [29]. This effect was also indirectly confirmed through the discoloration of phenol red in the SARS-CoV-2 media collected in the filter sample, due to the oxidation process (Fig 3A) [30]. Moreover, hydroxyl radicals can be generated in aerosols containing water molecules while passing through the plasma zone [31], which may have a significant viral inactivation effect. However, owing to experimental conditions, not only viruses contained in aerosols but also viruses collected in filter samples can be subsequently exposed to ozone; thus, further studies are required to verify whether the virus can be immediately deactivated in the process of passing through the DFD generator.

**Fig 4:**
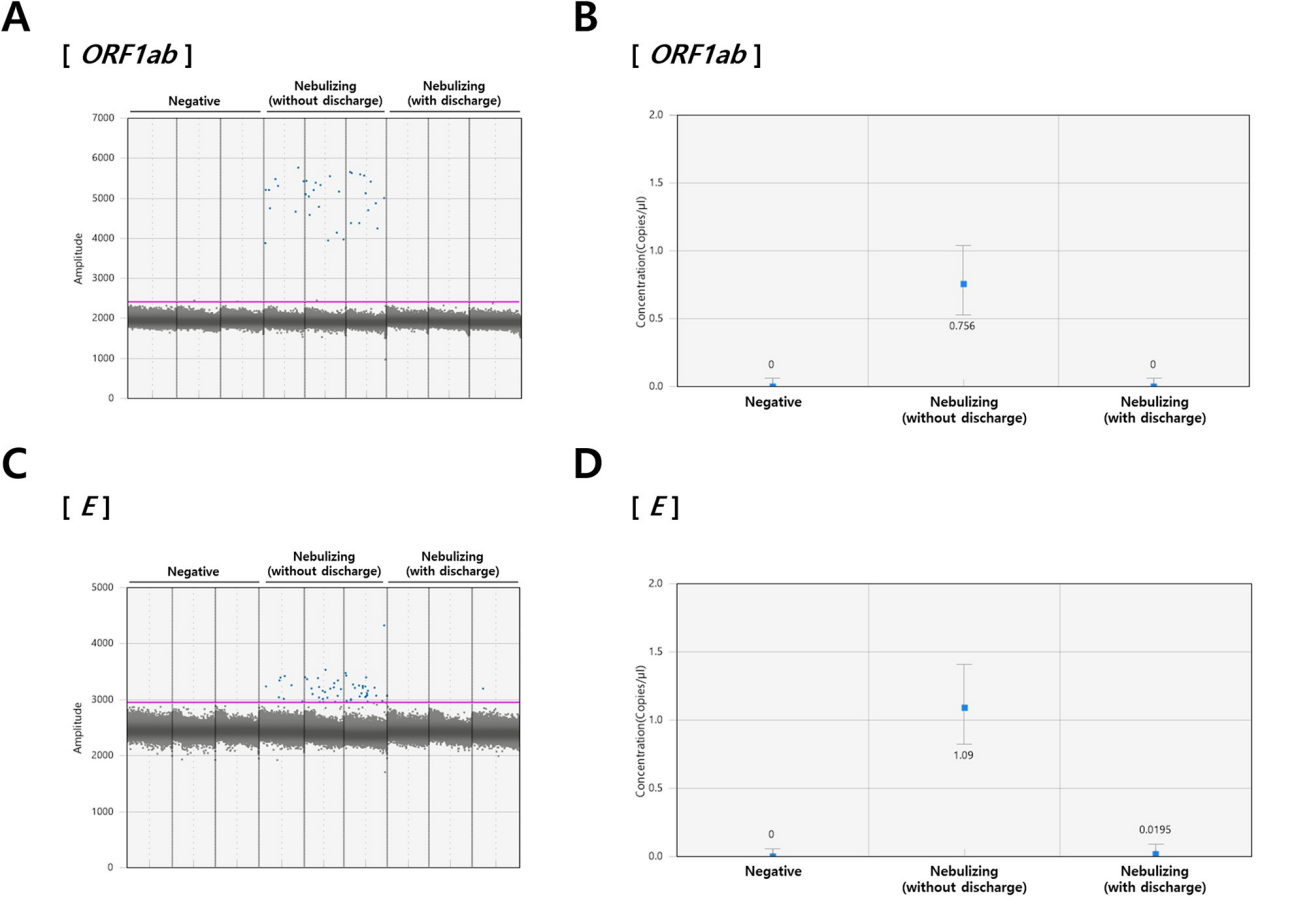
Quantification of SARS-CoV-2 viral genes. SARS-CoV-2 (A, B) *ORF1ab* and (C, D) *E* were detected in the nebulized samples using droplet digital polymerase chain reaction and quantified using the QuantaSoft software. (A, B) Each blue dot represents a droplet containing fluorescein-labelled DNA fragments. (C, D) Error bars indicate the 95% Poisson confidence intervals for each measurement.

*ORF1ab* and *E* are important factors influencing codons located in functionally important protein domains [32]; thus, they were selected as target genes to investigate the DFD-induced SARS-CoV-2 inactivation (Fig 4). Overall, the expression of both viral genes was found to be significantly reduced compared with that of the control group (Fig 4B and 4D). These results are consistent with a previous study [33] showing that ozone can contribute to the inactivation of SARS-CoV-2 by neutralizing *ORF1ab*, *E*, and *N* genes. Ataei-Pirkooh *et al*. [34] also demonstrated that ozone can disrupt the viral envelope, convert cysteine into cystine through the formation of a disulfide bond between adjacent viral particles, and eventually destroy the protein structure by releasing zinc ions from the viral protein structures. Therefore, DFD plasma has the potential to inactivate airborne SARS-CoV-2 by destroying its protein structure and inducing damage to various SARS-CoV-2 genes.

Since fine SARS-CoV-2 aerosol particles have an extensive exposure area and can be affected by various reactive species in the process of directly passing through the plasma zone, the virucidal potential may be superior compared with that under general antiviral experimental conditions (plasma treatment after surface inoculation). Therefore, it would be worthwhile to conduct a virucidal test for bioaerosols under lower ozone conditions, which could minimize the overestimation of ozone concentration or discharge conditions required to inactivate bioaerosols. In addition, the World Health Organization recommends that exposure to ozone concentrations above 0.1 ppm does not exceed an average of 8 h a day [35]; thus, it is necessary to properly control ozone emissions within the scope of safety regulations and apply them to air ventilation systems.

## Conclusion

DFD was found to be effective in immediately inactivating aerosolized SARS-CoV-2. Bioaerosols passing through the DFD showed a remarkable inactivation effect compared to surface ozone treatment in plaque assay and PCR analysis. In the future, studies on plasma application to inactivate aerosolized viruses need to be conducted in an environment where various factors, such as test space, diffusion dryer, discharge conditions, and flow rate, are sufficiently considered.

## Supporting information

S1 Fig Configuration of the air-passable dielectric filter discharge generator. (TIF)

S2 Fig Representative images of crystal violet-stained plaque assay plates. Vero 76 cells were inoculated with a SARS-CoV-2 culture. Plates were fixed three days after infection and stained with crystal violet. Plaques were counted to estimate the virus titer.
(TIF)
