# Supplementary figures and images for: Instant inactivation of aerosolized SARS-CoV-2 by dielectric filter discharge
Source: PLoS One. 2022 May 19;17(5):e0268049. doi: 10.1371/journal.pone.0268049 (PMC9119459; doi:10.1371/journal.pone.0268049)

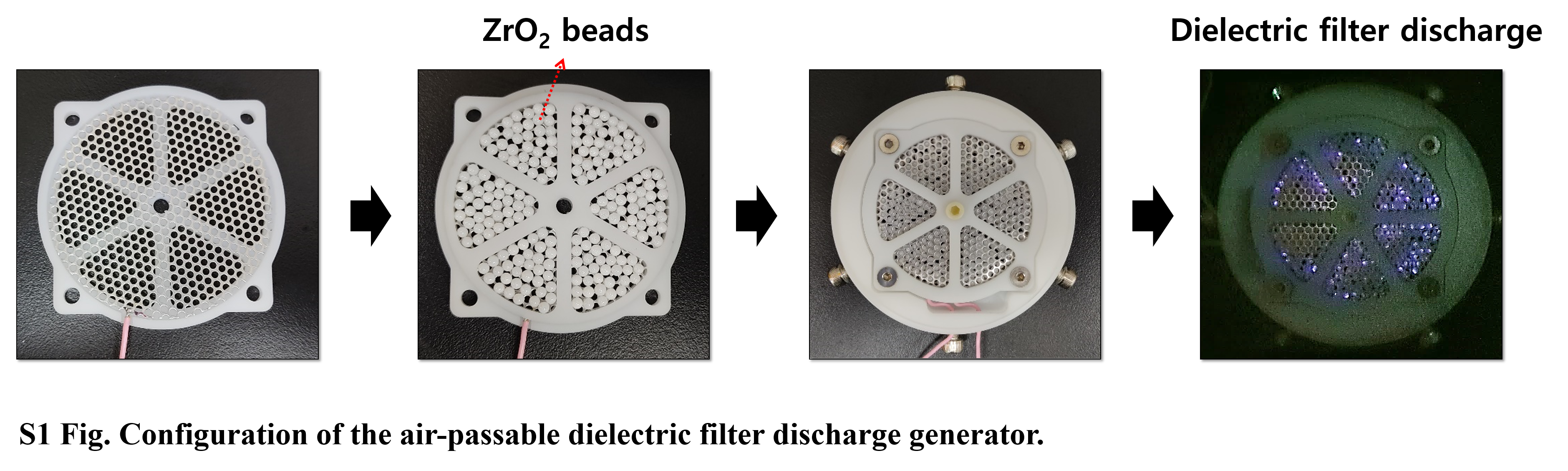

Supplement: S1 Fig — (TIF) [file pone.0268049.s001.tif]

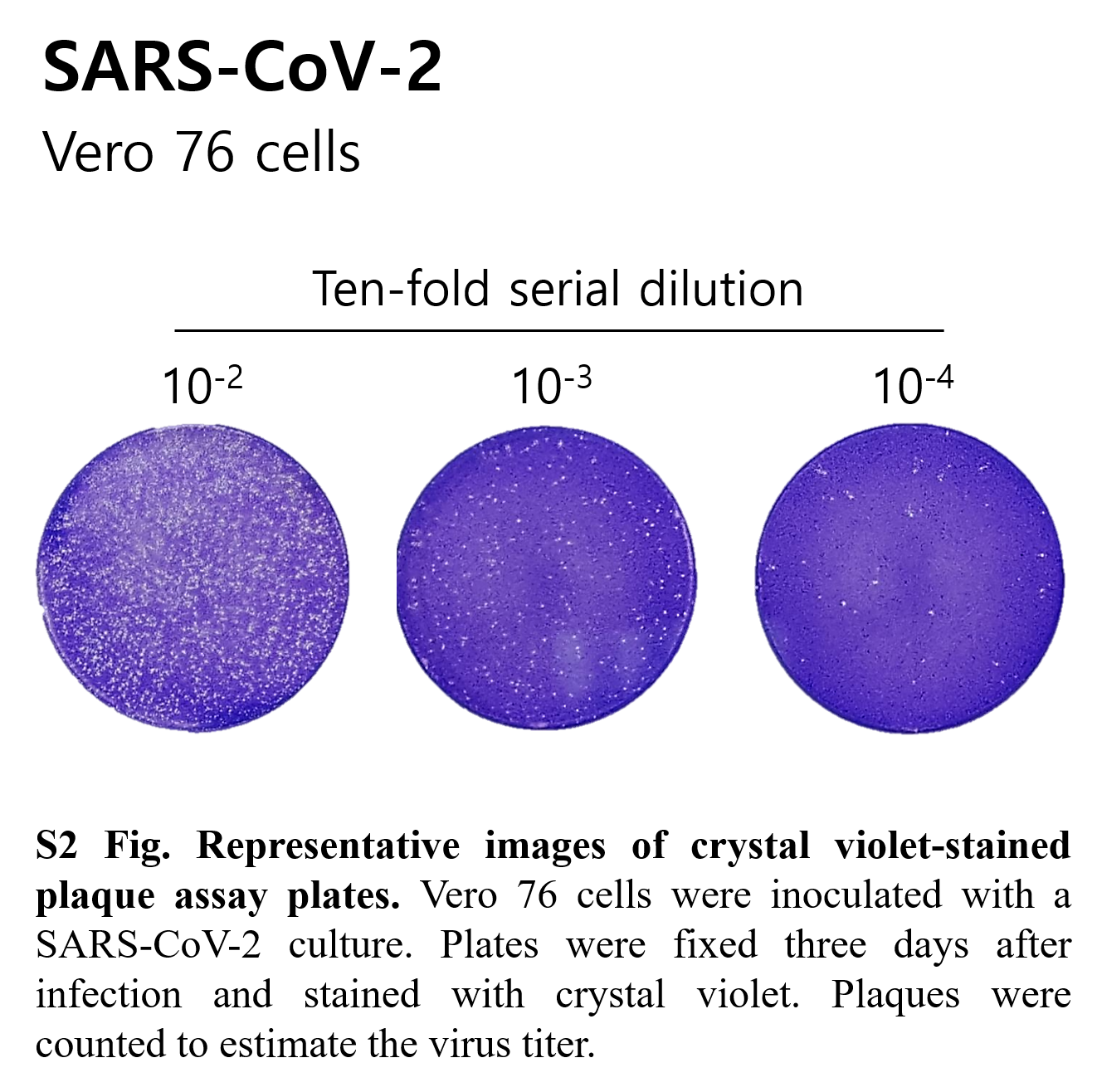

Supplement: S2 Fig — Vero 76 cells were inoculated with a SARS-CoV-2 culture. Plates were fixed three days after infection and stained with crystal violet. Plaques were counted to estimate the virus titer. (TIF) [file pone.0268049.s002.tif]
